# A mobile phone application for the assessment and management of youth mental health problems in primary care: health service outcomes from a randomised controlled trial of *mobiletype*

**DOI:** 10.1186/1471-2296-14-84

**Published:** 2013-06-19

**Authors:** Sophie C Reid, Sylvia D Kauer, Stephen J C Hearps, Alexander H D Crooke, Angela S Khor, Lena A Sanci, George C Patton

**Affiliations:** 1 Murdoch Childrens Research Institute, Melbourne, Australia; 2 Royal Children’s Hospital, Melbourne, Australia; 3 Department of General Practice, University of Melbourne, Melbourne, Australia; 4 School of Behavioural Science, University of Melbourne, Melbourne, Australia

## Abstract

**Background:**

GPs detect at best 50c of mental health problems in young people. Barriers to detecting mental health problems include lack of screening tools, limited appointment times and young people’s reluctance to report mental health symptoms to GPs. The *mobiletype* program is a mobile phone mental health assessment and management application which monitors mood, stress and everyday activities then transmits this information to general practitioners (GPs) via a secure website in summary format for medical review. The current aims were to examine: (i) *mobiletype* as a clinical assistance tool, ii) doctor-patient rapport and, iii) pathways to care.

**Methods:**

We conducted a randomised controlled trial in primary care with patients aged 14 to 24 years recruited from rural and metropolitan general practices. GPs identified and referred eligible participants (those with mild or more mental health concerns) who were randomly assigned to either the intervention group (where mood, stress and daily activities were monitored) or the attention-comparison group (where only daily activities were monitored). Both groups self-monitored for 2 to 4 weeks and reviewed the monitoring data with their GP. GPs, participants and researchers were blind to group allocation at randomisation. GPs assessed the *mobiletype* program as a clinical assistant tool. Doctor-patient rapport was assessed using the General Practice Assessment Questionnaire Communication and Enablement subscales, and the Trust in Physician Scale (TPS). Pathways to care was measured using The Party Project’s Exit Interview.

**Results:**

Of the 163 participants assessed for eligibility, 118 were randomised and 114 participants were included in analyses (intervention n = 68, attention-comparison n = 46). T-tests showed that the intervention program increased understanding of patient mental health, assisted in decisions about medication/referral and helped in diagnosis when compared to the attention-comparison program. Mixed model analysis showed no differences in GP-patient rapport nor in pathways to care.

**Conclusions:**

We conducted the first RCT of a mobile phone application in the mental health assessment and management of youth mental health in primary care. This study suggests that *mobiletype* has much to offer GPs in the often difficult and time-consuming task of assessment and management of youth mental health problems in primary care.

**Trial registration:**

ClinicalTrials.gov NCT00794222

## Background

Seventy-five percent of major mental disorders and alcohol or other substance misuse disorders have their onset prior to the age of 25 [1,2]. Adolescence is therefore clearly an ideal time for prevention and early intervention for mental illnesses. As General Practitioners (GPs) are the gatekeepers of mental health care [3], and most adolescents see a GP at least once a year [4], GPs are in an ideal position to identify young people at need or those who would benefit most from early intervention. Nevertheless, it is estimated that GPs are only identifying 50% of patients with mental health disorders [5,6]. Research suggests that this low rate of detection is most likely due to a lack of systematic approach to identifying at-risk patients and providing evidence-based treatments rather than a lack of skills on the part of the GP [5]. The majority of young people prefer to seek help from family and friends for mental health problems rather than general practitioners [7]. The main deterrents to seeking help from a GP were embarrassment or concern about what the GP might think of them [7,8]. Patient-doctor rapport plays a large role in a young person’s willingness to discuss general and mental health concerns with their GP [9] and necessarily better detection rates of youth mental health problems are associated with this rapport [6].

The high prevalence of mental health symptoms amongst young people, as well as their reluctance to raise these subjects [10], suggests a need for routine screening for mental health symptoms, as is standard practice for physical health symptoms [11]. In addition to improving the rate of detection of mental health symptoms, systematic measures increase a sense of partnership between patient and clinician, empower the patient to take control of their health, reduce feelings of helplessness and encourage patients to recognise early warning signs of relapse and to seek earlier intervention for future episodes [11,12]. Despite this need, GPs have only a limited amount of time to spend with their patients, with most appointments in Australia lasting only 15 minutes [13]. We recently developed and trialled a mobile phone application, called “Mobile Tracking Young People’s Experiences” (*mobiletype*). The aim of the *mobiletype* program is to support doctors and provide information about young people’s general and more specific mental health functioning to assist in determining appropriate treatment approaches and management plans. It is a youth-friendly, time-saving mental health assessment which can be used at any time point within the patient care trajectory [14-16]. Mobile phones are ideally suited to the busy primary care environment as they are ubiquitous (over 95% of young people own one), ownership crosses socioeconomic and geographic boundaries, and have the ability to capture a large amount of information with minimal outlay of GP time. Furthermore, young people often report feeling more able to disclose sensitive information via technological means rather than face to face communication [17].

The *mobiletype* program monitors a young person’s mood, stress, coping strategies and daily activities a number of times per day, and their eating, sleeping, exercise patterns and alcohol and cannabis use once per day. This information is then uploaded to GPs, via a secure website and displayed in summary reports for review, in a manner similar to a pathology or radiology report [14]. Our studies suggest that young people will monitor their mental health symptoms for the purpose of reviewing this data with their doctor, that both doctor and young person find this a beneficial way of communicating information about mental health, and that the *mobiletype* program assisted the doctor to understand the young person better [14,18].

The overall aim of this study was to investigate, via a randomised controlled trial, a number of suggested benefits found in our pilot studies of the *mobiletype* program [14-16,19], being the collection of clinically useful data in a time efficient manner, increased doctor-patient rapport, and subsequently, better pathways to care due to more thorough assessment of patient. In this RCT we were interested in testing the utility of the *mobiletype* program in the real world primary care setting and therefore sought to have broad inclusion criteria such that the findings may best approximate the nature of the primary care setting. This paper reports on the health service outcomes of the RCT (mental health outcomes can be found in BMC Family Practice [14]), the following questions were investigated: i) To what extent does the *mobiletype* function as a clinical assistance tool for GPs in their assessment and management of mental health symptoms in young people compared with an attention-comparison (described in Methods), ii) does the *mobiletype* program enhance doctor-patient rapport compared to an attention-comparison, iii) and does the *mobiletype* program assist in leading young people into pathways to care for mental health symptoms compared to an attention-comparison?

## Methods

As the present reports on the secondary outcomes of a larger RCT, a full description of methodology is reported elsewhere [14].

### Study design

The data presented here are the secondary outcome data from the *mobiletype* randomised controlled trial conducted from 2009 to 2011. This was a multi-centre, multi-regional, stratified (according to region), single blind, attention-controlled study with balanced (1:1) individual randomisation into parallel-groups. This study was conducted in Victoria, Australia in a manner to allow for strict adherence to CONSORT reporting guidelines [20].

### Recruitment

#### General practitioners

All general practitioners in the Goulburn Valley Region and the Albury/Wodonga Region were invited to participate in the study via the Regional Division of General Practice (support units that service clinical practices within a region). GPs in Melbourne were recruited via the local Divisions of General Practice. Clinics were targeted that listed an interest in adolescent health on the Melbourne General Practice Network (http://www.mgpn.com.au). Participating GPs were trained in using the *mobiletype* website and the study procedure, and provided a thorough range of clinical support and literature. Continuing professional development quality assurance points were available to GPs for their participation in the study. Weekly reminder faxes were sent to all participating GPs and fortnightly phone calls to the GPs clinic were made to remind doctors of the study and to provide an update on recruitment to the study.

#### Young people

To best approximate the real world primary care setting, the following participant inclusion criteria were set: (1) aged 14 to 24 years, (2) speak proficient English and (3) have a mild or more severe emotional/mental health issue as assessed by their GP, or indicated by a K10 Symptom score greater than 16 [21]. Participants were excluded if they had a severe psychiatric or medical condition that prevented them from complying with either the requirements of informed consent or study protocol (i.e. current psychosis).

#### The mobiletype programs

Version 4 of the *mobiletype* program was used as the intervention in this study which was created using Java Platform, Micro Edition, in-house by the Murdoch Childrens Research Institute. This program was written for use with multiple models of mobile phones and firmware. For the purposes of this trial participants were lent a study mobile phone with either the *mobiletype* intervention or comparison program downloaded onto it.

Participants were prompted to complete a *mobiletype* entry by an auditory signal/beep emitted from the mobile phone at random intervals in the blocks outlined in Table 1, with a reminder signal after 5 minutes. Participants were also able to complete the program any time and were able to complete an entry between 10 pm and 8 am although no trigger was sent at this time. The night time entry (00:00–08:00) consisted of the same questions as the afternoon questions as shown in Table 1. Each report took approximately 1–3 minutes to complete.

**Table 1:** **Modules included in each block of the *mobiletype *intervention and comparison programs**

	**Intervention**				**Attention Comparison**			
	**Morning**	**Noon**	**Afternoon**	**Evening**	**Morning**	**Noon**	**Afternoon**	**Evening**
	**08:00–10:59**	**11:00–15:29**	**15:30–19:59**	**20:00–00:00**	**08:00–10:59**	**11:00–15:29**	**15:30–19:59**	**20:00–00:00**
*MODULE*								
Current Activity	X	X	X	X	X	X	X	X
Stress	X	X	X	X				
Mood	X	X	X	X				
Alcohol Use		X						
Cannabis Use		X						
Sleep	X				X			
Diet				X				X
Exercise				X				X

#### Intervention group

The intervention group monitored themselves using the complete *mobiletype* program which assessed 8 areas of functioning as developed in previous *mobiletype* studies [16,19], consisting of current activities, location, companions, mood, recent stressful events, responses to stressful events, alcohol use, cannabis use, quality and quantity of sleep, and quantity and type of exercise, and diet (meals, snacks, “junk-food,” and “soft-drinks” consumed). Participants who indicated they were at risk of self-harm or suicide activated the program’s high-risk alert, which would automatically send an SMS to our on call psychologist/phone counsellor. The psychologist would then call the young person and assess the risk of self-harm and alert the participant’s local community assistance team if necessary. The time of day each module assessing the eight areas was delivered varied as displayed in Table 1. Daily activities, stress and mood were assessed each time the participant completed the program, diet, exercise, sleep, alcohol and cannabis use were assessed once a day at different times.

#### Attention-comparison group

The attention comparison group was designed to provide a data collection process similar to the intervention group by controlling for the amount of time spent engaged in the research methodology and the attention given to them by health care professionals and research staff [22]. The comparison group monitored themselves using an abbreviated version of the *mobiletype* program which assessed only current activities, location, companions, quality and quantity of sleep, and quantity and type of exercise, and diet. Importantly, the modules pertaining mental health as per Table 1 (i.e. mood, stress, alcohol and cannabis use) were removed. Daily activities were assessed at each time point and diet, exercise and sleep were assessed once a day at different time points.

#### Summary reports

Data collected by the *mobiletype* program (intervention and comparison) on the mobile phone were sent via SMS to a secure website constructed and hosted by MCRI, where it was automatically collated. Each area of assessment was displayed in graphs (i.e. daily mood graphs) or in tables (i.e. daily alcohol intake). An individualised summary report of the data was written following structured prescriptive guidelines by the first author (registered psychologist), or the second author under the supervision of the first author. The intervention summary reports consisted of mood, stress and coping, maintaining wellbeing and useful resources and recommendations for the intervention group. The comparison group also received individualized summary reports consisting of maintaining wellbeing (about their sleep, daily activities, diet, and exercise) and useful resources and recommendations. A copy of the summary report has been published previously [14].

### Outcome measures

A full description of all outcome measures are reported in the main RCT outcome paper [15]. The present paper focuses on a selection of participant and GP variables pertaining to *mobiletype* as a clinical assistance tool, GP/patient rapport, and pathways to care.

#### Clinical assistance tool

After the post-test medical review GPs completed a series of 5-point Likert scale items (0 = Very poor/unhelpful to 4 = Very well/helpful), concerning the extent to which *mobiletype* assisted both the GP and the patient understand the patient’s current functioning, and was helpful with diagnosis, communication, maintaining trust, understanding patients’ specific medical problem, understanding patient’s mental health problems, knowledge about the patient (i.e. lifestyle, behaviours, etc.), and gaining a good overall picture of their patient’s current functioning. At pre-test and post-test, GPs answered an adapted version of the SHO Appraisal Form [23]. This measure requires GPs to rate how confident they were in their diagnosis, communication, maintaining trust, understanding patients’ specific medical problem, understanding patient’s mental health problems, knowledge about the patient (i.e. lifestyle, behaviours, etc.) on a series of 4-point Likert scale (0 = Not confident, 1 = Lacking confidence, 2 = Confident, 3 = Very confident). These items were summed into a GP Confidence scale, and internal consistency was tested by Cronbach’s alpha [α] [24] and was 0.93. GPs completed these questionnaires for each participant after their post-test medical review regardless of group allocation, and the questionnaires did not specify which group the patient was allocated to.

#### Doctor patient rapport

Doctor-patient rapport was measured from the patients’ perspective with the General Practice Assessment Questionnaire (GPAQ) Communication and Enablement subscales [25], and the Trust in Physician Scale (TPS) [26] which were completed at pre-test, post-test and 6 weeks post-test. Internal consistency was again confirmed by Cronbach’s alpha (GPAQ Communication α = 0.96; GPAQ Enablement α = 0.88; TPS α = 0.71).

#### Pathways to care

The Party Project’s Exit Interview [27] was completed by the participants and assessed pathways to care being if the participant was i) prescribed medications, ii) referred to a health professional, ii) referred for further testing, scans and/or X-rays, or iv) provided other advice and psychoeducation regarding mental health during the most recent medical review. These dichotomous items were summed into a Pathways to Care scale (range: 0–4).

### Sample size

Recruitment of 200 participants was anticipated from 10 general practices. This sample size was based upon Cohen’s [28] statistical testing for multiple regression with two independent variables (to account for the mediating variable and the outcome) to detect a medium effect with 80% power and a probability of a type I error of .05. A medium effect size was selected as this was thought to be clinically significant. The anticipated sample size of 200 was not met due to delays in recruitment during school holidays and the H1N1 influenza pandemic [29]. As a result, a deadline was set for cessation of recruitment, and a total of 118 participants were recruited.

### Randomisation

Participants were randomised to either i) the *mobiletype* monitoring intervention program group or ii) the comparison program group; both groups also received care as usual. Randomisation was carried out by an in-house computer programmer who had no research or recruitment role in the study using random seed generation at the individual-level and stratified according to area (Melbourne, Goulburn Valley, and Albury/Wodonga). Study mobile phones were allocated ID numbers within areas (i.e. Melbourne01, Melbourne02) and either the intervention or comparison *mobiletype* program was loaded consecutively in a blinded fashion according to the programmer’s concealed randomisation list. The randomisation list was constructed for 100 Melbourne, 50 Goulburn Valley and 50 Albury/Wodonga participants. Researchers, participants, and GPs were blind to randomisation pre-test. GPs and participants became aware of the group allocation at the post-test when the summary reports were reviewed. This study had Royal Children’s Hospital Human Research Ethics Committee approval (HREC: 28113), was registered in ClinicalTrials.gov (Reference: NCT00794222) and adhered to ethical guidelines for data collection, storage and reporting.

### Procedure

#### Recruitment

In addition to treatment as usual, GPs screened their patients for eligibility to the study, organised an appointment for interested participants with a research. Participants met with a *mobiletype* research assistant within five days of referral to learn the study process, complete consent forms, the pre-test questionnaire package, review the *mobiletype* program and other features of the phone and complete a practice entry of the *mobiletype* program. Participants were provided with a study manual that described the research procedure and offered trouble-shooting tips.

#### Mobile phone monitoring period

All participants borrowed a Sony Ericsson Z750i mobile phone containing the *mobiletype* program for the study period. Information regarding the development and testing of the *mobiletype* program has been previously published [16]. Participants were requested to complete at least two *mobiletype* entries a day until they returned for their medical review in 2–4 weeks; participants and GPs were advised that 2–4 weeks was the ideal monitoring period. Participants were given a SIM card containing $30 in credit as partial reimbursement for their time and phone credit used.

#### Post-test review, 6-week post-test, and 6 month post-test assessments

Upon completion, participants reviewed the self-monitoring data with their GP on the *mobiletype* website. Young people completed a post-test assessment immediately following this appointment, again at six weeks and six months after this post-test review (six month post-tests not included in the current analysis). GPs completed a post-test questionnaire immediately after the appointment. Questionnaires were completed online, over the phone with a research assistant, or via a mailed hardcopy survey. Participants were given a $20 gift card for each follow up survey completed (maximum of $60 for all questionnaires completed).

### Analyses

Demographic data and missingness (to satisfy the MAR assumption for random-effects modeling) are reported elsewhere [14]. For data measured at one time point (the GP Clinical assistance items) independent t-tests were employed to compare mean differences of complete cases between groups. For data measured at two time points (GP Confidence scale) regression analyses were conducted, in which the difference between groups were explored after adjusting for pre-test scores [30].

Finally, for data measured at three timepoints (GPAQ scales, TPS and Pathways to Care scale), mixed model analysis was employed, using the MIXED procedure in SPSS (consistent with methodology carried out in the primary outcomes article [14]). Contrary to repeated measures analysis of variance, mixed model methods analyse all available data without any data loss [31]. All mixed model analyses employed a restricted maximum likelihood estimation method, included subject id as a random effect and survey time to create individual random slopes.

Clustering at the GP level was considered and tested, but due to the number of GPs involved and the relatively small clusters within GPs, clustering at the GP level did not significantly contribute to the model or provide a better approximation of the intraclass correlation coefficient than at the individual level.

## Results

In sum, the mean participant age was 18.1 years (SD = 3.2), and the majority were female (71.9%), students (66.7%) and did not identify with an ethnic background (87.3%). No significant demographic differences were found between groups, and the MAR assumption was upheld. Rural areas were purposefully overrepresented in this study with 26 different practices in the three recruitment areas: 12 in greater Melbourne, 7 in Wodonga and 7 in the Goulburn Valley.

To evaluate the effectiveness of the *mobiletype* program, independent *t*-tests compared responses of GPs’ with participants in the intervention group to those with patients in the comparison group. The results can be seen in Table 2.

**Table 2:** Comparison of observed group means and standard deviations of GP clinical assistance items

		**Comparison**		**Intervention**	***p***	***d***
	**n**	**M (SD)**	**n**	**M (SD)**		
*How well did the* mobiletype *information assist with…*						
Gaining picture of patient’s current functioning?	39	2.62 (0.71)	56	3.00 (0.85)	.023	0.48
Helping patient better understand current situation/functioning?	39	2.56 (0.88)	56	2.75 (0.94)	.333	0.20
*To what extent was the* mobiletype *program helpful with.*						
Medication?	36	2.33 (1.04)	54	2.89 (1.00)	.013	0.55
Alternative treatment not involving medication?	37	2.76 (1.04)	55	3.11 (0.94)	.094	0.36
Referrals?	37	2.35 (0.98)	50	2.90 (0.99)	.012	0.56
*To what extent was the* mobiletype *program helpful with…*						
Diagnosis?	36	2.47 (1.16)	55	3.18 (0.92)	.002	0.69
Communication?	37	2.92 (1.14)	54	3.39 (0.90)	.031	0.47
Maintaining trust?	37	2.95 (1.10)	54	3.09 (0.98)	.506	0.14
Patient’s specific medical problem?	36	2.42 (1.08)	48	2.96 (1.01)	.021	0.52
Patient’s mental health problems?	36	2.81 (1.12)	55	3.36 (0.91)	.011	0.56
Knowledge about patient?	37	3.27 (1.02)	53	3.43 (1.03)	.458	0.16

*p* = *t*-test significance.

*d* = Effect size; Cohen’s *d.*

Results indicate that when participants completed the intervention *mobiletype* program, their GPs found that the *mobiletype* program significantly assisted them in gaining a picture of their patient’s current functioning (*p* = .023, effect size [*d*] = 0.48) than when participants completed the attention comparison program. Moreover, GPs reported that the *mobiletype* program was significantly more helpful in deciding about medication (*p* = .013, *d* = 0.55) and referrals (*p* = .012, *d* = 0.56) compared to the attention comparison. Finally, GPs indicated that the *mobiletype* program helped in their diagnosis (*p* = .002, *d* = 0.69) and communication (*p* = .031, *d* = 0.47), as well as their patient’s specific medical (*p* = .023, *d* = 0.52) and mental health problems (*p* = .011, *d* = 0.56), significantly more than when using the attention comparison.

The difference between groups at post-test in the GP Confidence scale (Cronbach’s alpha [α] = 0.93) were tested by regression analysis, adjusting for pre-test GP Confidence scale score. After adjusting for baseline values, the difference between intervention and comparison group’s doctors confidence was not significant (β = 1.05, *p* = .131).

Three rapport-based scales - GPAQ Communication and Enablement subscales, and TPS were tested by way of mixed model analysis. Observed means and standard deviations of these scales can be seen in Table 3. The GPAQ Communication scale analysis returned a nonsignificant group x time interaction effect, *F*(2, 77.74) = 0.96, *p* = .386 and no significant main effect for group *F*(1, 101.87) = 0.89, *p* = .347, or time *F*(2, 77.74) = 0.09, *p* = .917. Similar results were found for the GPAQ Enablement subscale with nonsignificant group x time interaction, *F*(2, 70.85) = 0.18, *p* = .839, and main effects for group, *F*(1, 97.76) = 0.00, *p* = .992, and time, F(2, 70.85) = 2.73, *p* = .072. The final rapport-based scale, TPS (α = 0.71), also returned nonsignificant results, with no significant interaction *F*(2, 63.90) = 1.00, *p* = .375, or main effects for group, *F*(1, 105.52) = 0.34, *p* = .562 or time, *F*(2, 63.90) = 0.46, *p* = .631.

**Table 3:** Observed group means and standard deviations of the GP confidence scale and the doctor-patient rapport scales

	**Comparison**		**Intervention**	
	**n**	**M (SD)**	**n**	**M (SD)**
GP Confidence*				
Pre	34	14.11 (4.57)	50	15.48 (3.22)
Post	34	12.56 (4.13)	50	13.14 (4.29)
*GPAQ Communication*^*+*^				
Pre	44	76.6 (17.4)	66	76.8 (19.5)
Post	32	80.2 (18.5)	47	74.8 (25.3)
6-Week	34	76.8 (20.2)	41	76.5 (20.9)
*GPAQ Enablement*^*+*^				
Pre	42	42.9 (27.1)	62	41.8 (31.9)
Post	30	52.5 (32.4)	42	50.6 (31.3)
6-Week	30	43.9 (35.7)	36	51.9 (39.8)
*TPS*^*+*^				
Pre	42	73.0 (12.7)	62	74.7 (16.3)
Post	32	74.6 (17.6)	46	71.0 (20.4)
6-Week	35	74.9 (15.2)	45	73.7 (16.7)

* = OLS regression analysis.

^+^ = Mixed model analysis.

**Figure 1:**
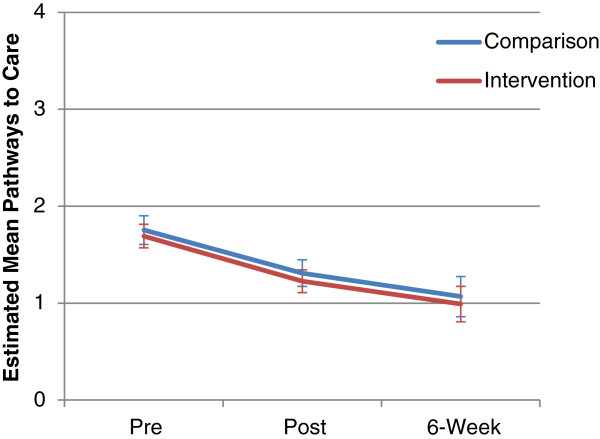
Estimated means and standard errors of the Pathways to Care Scale between groups over the three survey measurement periods.

The summed Pathways to Care scale was also tested by mixed model analysis. Results from the main effects showed a significant effect of time, *F*(2, 77.96) = 13.83, *p* < .001, but no significant group main effect, *F*(1, 86.01) = 0.25 *p* = .618. Furthermore, the interaction effect of group x time was not significant, *F*(2, 77.96) = 0.01, *p* = .995. Estimated means (and standard errors) from the mixed model analysis are shown in Figure 1, and suggest that for the majority of participants, most pathways to care were implemented at pre-test.

## Discussion

The aim of this RCT was to examine the health service outcomes of the *mobiletype* program in primary care, in particular the extent to which the *mobiletype* could provide clinical assistance, enhance doctor-patient rapport and lead to pathways to care. GPs reported that the *mobiletype* program assisted them to understand their patients’ current functioning, mental and physical health problems and assisted them with diagnoses, communication, medication, referrals and significantly more than the attention comparison. These findings suggest that a mobile phone monitoring program which captures and summarises detailed, specific mental health and more general health information in a time-efficient manner may assist GPs in their management of youth mental health problems. Interestingly, whilst there was reasonably high compliance with the monitoring protocol both in this study [14] and our previous studies [16,18,19], and our pilot study suggested that young people thought that using the *mobiletype* program was a useful way of transferring information between patient and doctor, there was no significant effect of the *mobiletype* program on doctor-patient rapport from the young person’s perspective when tested across time and compared to an attention comparison program. Finally, there was no significant effect of the *mobiletype* program on pathways to care, rather as reported in the mental health outcomes paper [14] it appeared that GPs conducted a mental health intervention at pre-test, implementing on average two pathways to care for most participants.

Assisting GPs in the assessment and management of youth mental health problems is critical [4,12], and the *mobiletype* program via its design offers a solution to a number of the doctor and patient barriers in detecting and managing mental health. *mobiletype* is systematic in assessment, time-efficient, provides detailed mental and general health information, generally well-received by patients [14], low-cost, and uses a communication technology that crosses traditional geographic and socioeconomic barriers. The finding that GPs found it of assistance in overall assessment, determining diagnoses, medications and referrals, and assisted in communication between GP and patient is further support for the utility of the *mobiletype* program as clinical assistance tool and adds to the growing literature on the acceptability and benefits of computerised screening in primary care to assist GPs [32,33].

It was surprising, given our pilot study findings [18] that using the *mobiletype* program did not assist doctor-patient rapport significantly more than attention comparison program. Nevertheless, both the *mobiletype* and the attention comparison program included monitoring and provision of feedback for doctors and participants to review. The review of personal monitoring data was the process that we hypothesised would lead to increased doctor-patient rapport, and hence a wait-list control group who did not monitoring may have been more appropriate comparison group to test this hypothesis. In addition, the participants ratings at pre-test of their satisfaction with their doctors was quite high, and anecdotally we found that many of the doctors volunteering for this study were experienced in working with young people and/or had an interest in mental health. Thus a ceiling effect may have occurred in terms of the ratings of doctor patient-rapport.

Finally, we did not find that the *mobiletype* program lead to a difference in the pathways to care compared to the attention comparison group. The significant main effect for time, suggested that GPs were unexpectedly acting to manage mental health during the pre-test review in two ways for the majority of participants, without waiting for further information from the *mobiletype* monitoring data, summary report and recommendations, and then were less likely to implement further pathways to care at post-test and 6-week post-test. As discussed in the mental health outcomes paper [14] participating GPs may have felt the need to manage mental health symptoms when they first present rather than wait for further data as the general rate of return to follow up review appointments can be unpredictable [9,34].

### Limitations

Whilst the GPs rated the *mobiletype* program as of greater clinical assistance than the attention comparison program, these results came from a onetime measurement point. The current study failed to find changes across time in GP confidence or doctor-patient rapport, and it is possible that our measures of GP confidence and doctor-patient rapport were insensitive to the changes that may have occurred. Moreover, our pre-test rapport and GP confidence scores were relatively high, also suggesting a possible ceiling effect that decreased the likelihood of improving these scores. An alternate reason for the lack of significance could be that study may have failed to attract doctors who are most in need of clinical assistance tools for the detection and assessment of mental health in young people. Research trials relying on volunteer participation tend to attract those who are interested and motivated by the topic of the research, in this case youth and mental health.

The inclusion of a wait-list control or “usual medical care only” group in this study would have allowed for comparison and testing of the doctor-patient rapport hypothesis, nevertheless, this was not the main hypothesis of the study. Finally, further limitations are detailed in the mental health outcomes paper [14], which include: a cluster randomised controlled trial in which GPs rather than patients were randomised may have been more appropriate but was rejected as it would be logistically impossible for GPs and participants to remain blinded in the current study; participant heterogeneity in illness type, severity and familiarity with their GP due to broad inclusion criterion needed in an effectiveness trial is likely to have reduced the overall power of the study; and the random outcomes of uneven groups due to early cessation of recruitment is also likely to have reduced the power of study.

## Conclusions

We conducted the first RCT of a mobile phone application in the mental health assessment and management of youth mental health in primary care. This study suggests that *mobiletype* has much to offer GPs in the often difficult and time-consuming task of assessment and management of youth mental health problems in primary care.

## Competing interests

There are no competing interests for any authors in this publication.

## Authors’ contributions

SCR conceived of, designed, and implemented the study, she also participated in the analyses and drafted the manuscript. SK participated in the conception, design, and implementation of the study, participated in the analyses and contributed to the drafting of the manuscript. SJCH conducted the analyses and participated in drafting the manuscript. AHDC coordinated the implementation of the study and contributed to drafting the manuscript. ASK contributed to the design of the study, collected data for the study and contributed to drafting the manuscript. LAS and GCP both contributed to the design of the study and drafting of the manuscript. All authors read and approved the final manuscript.

## Pre-publication history

The pre-publication history for this paper can be accessed here:

http://www.biomedcentral.com/1471-2296/14/84/prepub
